# Supplementary material for: Palmitoylation Is Indispensable for Remorin to Restrict Tobacco Mosaic Virus Cell-to-Cell Movement in Nicotiana benthamiana
Source: Viruses. 2022 Jun 17;14(6):1324. doi: 10.3390/v14061324 (PMC9227848; doi:10.3390/v14061324)
Supplement: Supplementary file 1 [file viruses-14-01324-s001.zip › viruses-1738699-supplementary.pdf]

Supplementary Materials:

Percent Identity

	**Divergence**	**1**	**2**	**3**	**4**	**5**	**6**	**7**	**8**	**9**	
NbREM1.1	**1**		**98.6**	63.0	48.1	47.2	45.8	42.6	45.4	45.8	**1**
NbREM1.2	**2**	2.8		63.9	48.6	47.7	46.8	43.1	46.8	46.3	**2**
NbREM1.3	**3**	50.7	49.0		57.9	56.9	46.8	52.3	46.3	48.1	**3**
NbREM1.4	**4**	84.9	83.6	61.0		**97.2**	47.2	63.9	46.8	60.6	**4**
NbREM1.5	**5**	87.6	86.2	63.0	2.8		46.8	63.0	46.3	60.6	**5**
NbREM1.6	**6**	91.7	88.9	88.9	87.6	88.9		43.5	**91.7**	45.8	**6**
NbREM1.7	**7**	102.2	100.6	73.9	49.0	50.7	99.1		44.0	54.2	**7**
NbREM1.8	**8**	93.1	88.9	90.3	88.9	90.3	8.9	97.5		44.9	**8**
NbREM1.9	**9**	91.7	90.3	84.9	55.2	55.2	91.7	69.4	94.6		**9**
		**1**	**2**	**3**	**4**	**5**	**6**	**7**	**8**	**9**	

Figure S1. Pairwise identity analyses of the nine remorin proteins of the *N. benthamiana* group 1 remorin family by Clustal W method.

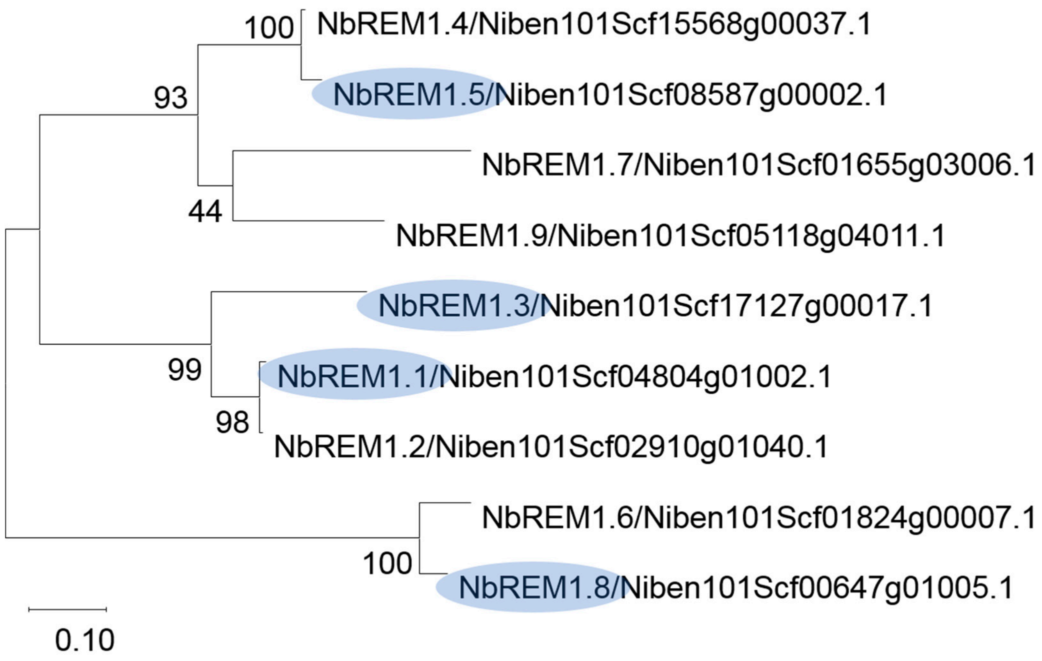

Figure S2. Evolutionary analysis of the nine remorin proteins of the *N. benthamiana* group 1 remorin family (NbREMs) by maximum likelihood method. The evolutionary history was inferred by using the Maximum Likelihood method and JTT matrix-based model with 9 NbREMs full-length proteins sequences. The tree with the highest log likelihood is shown. The percentage of trees in which the associated taxa clustered together is shown next to the branches. Initial tree(s) for the heuristic search were obtained automatically by applying Neighbor-Join and BioNJ algorithms to a matrix of pairwise distances estimated using the JTT model, and then selecting the topology with a superior log likelihood value. The tree was tested via bootstrap analysis (1,000 replicates) and was drawn to scale, with branch lengths measured in the number of substitutions per site. Evolutionary analyses were conducted in MEGA X. Blue box marks four NbREMs homologs used for assay in this study.

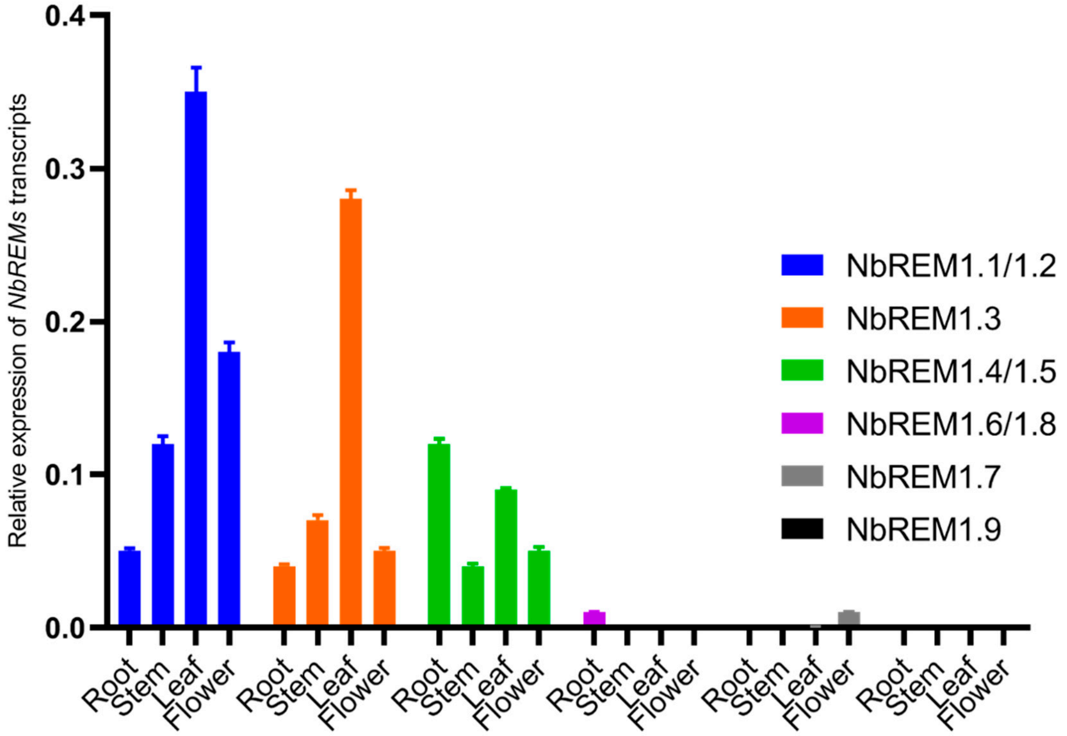

Figure S3. Detection of the relative mRNA expression of the nine remorin proteins of the *N. benthamiana* group 1 remorin family by RT-qPCR in different organs of *N. benthamiana*.

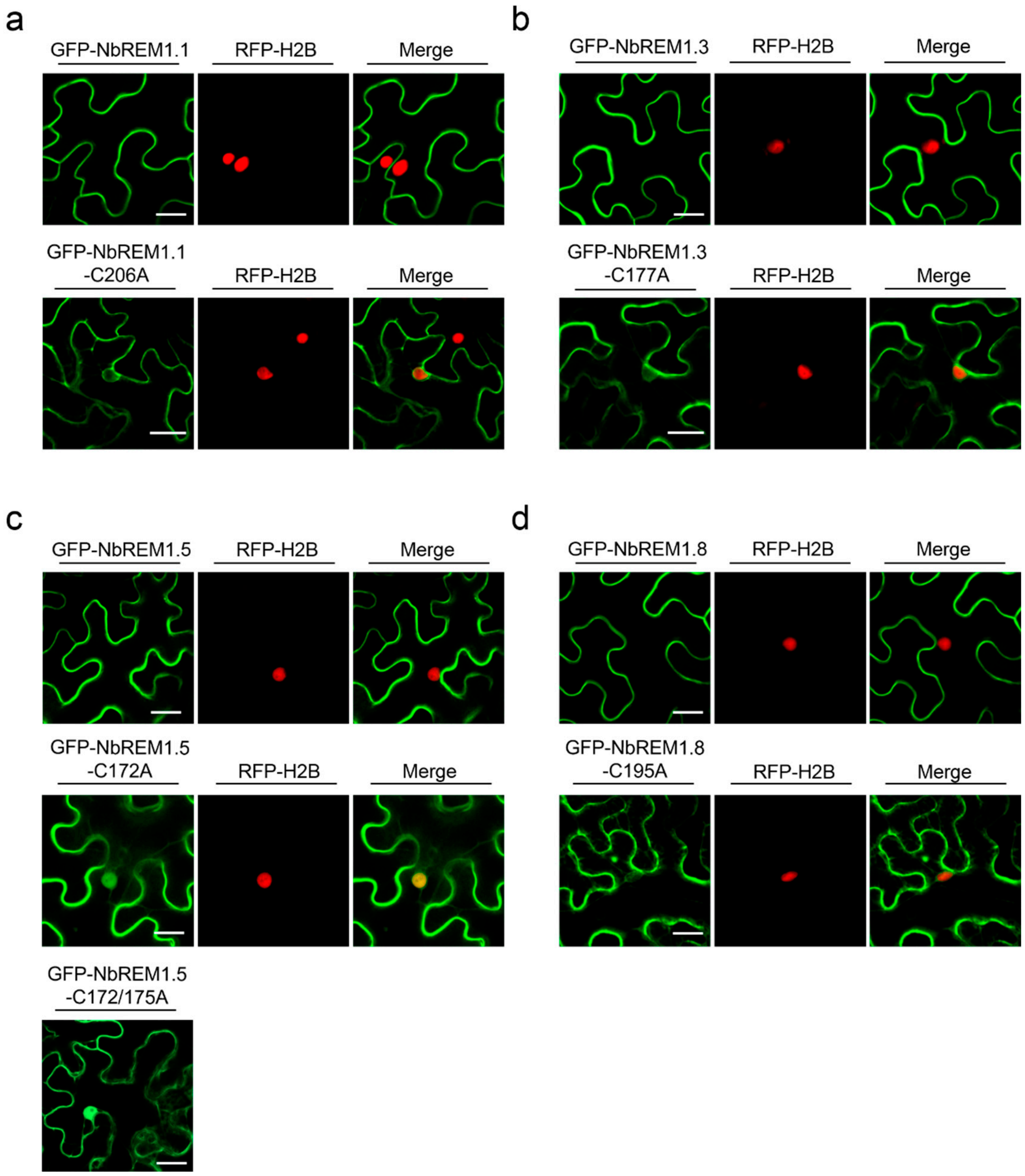

Figure S4. Localization of NbREMs and its respective palmitoylation-defective mutants in *N. benthamiana* epidermal cells. **(a)** NbREM1.1. **(b)** NbREM1.3. **(c)** NbREM1.5. **(d)** NbREM1.8. GFP-NbREMs and its palmitoylation-defective mutants were transiently expressed in transgenic *N. benthamiana* expressing H2B-RFP which the nuclei was marked by red fluorescence. Confocal images were taken at 48 hpi. Bar, 20 μm.

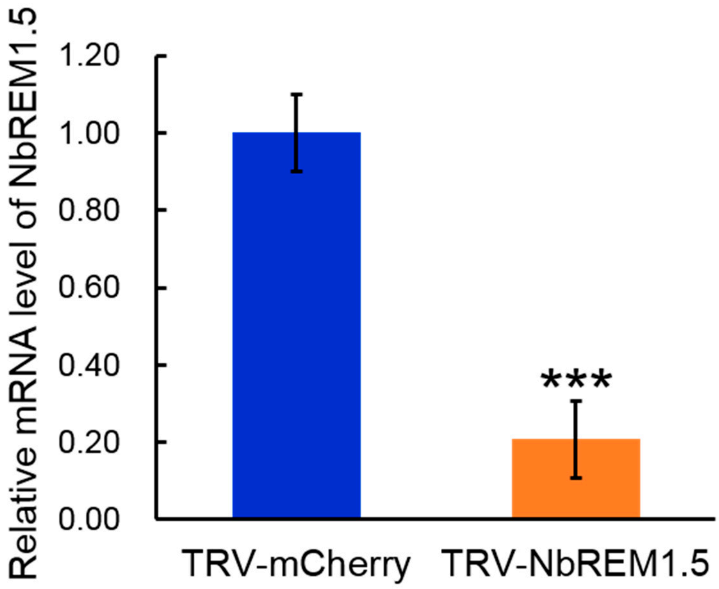

Figure S5. Knock-down of NbREM1.5 in *N. benthamiana* plants by tobacco rattle virus (TRV)-based gene silencing. A 350-bp *NbREM1.5* or *mCherry* cDNA fragment was cloned into TRV-RNA2 to generate TRV-RNA2::*NbREM1.5* or TRV-RNA2::*mCherry* used for silencing of NbREM1.5 in *N. benthamiana* or as a negative control. RT-qPCR was used to detect the relative mRNA level of NbREM1.5 in TRV-mCherry and TRV-NbREM1.5 inoculated *N. benthamiana* plant leaves at 10 dpi. Asterisks mark significant differences according to two-tailed Student's *t*-test; ***$P < 0.0001$.

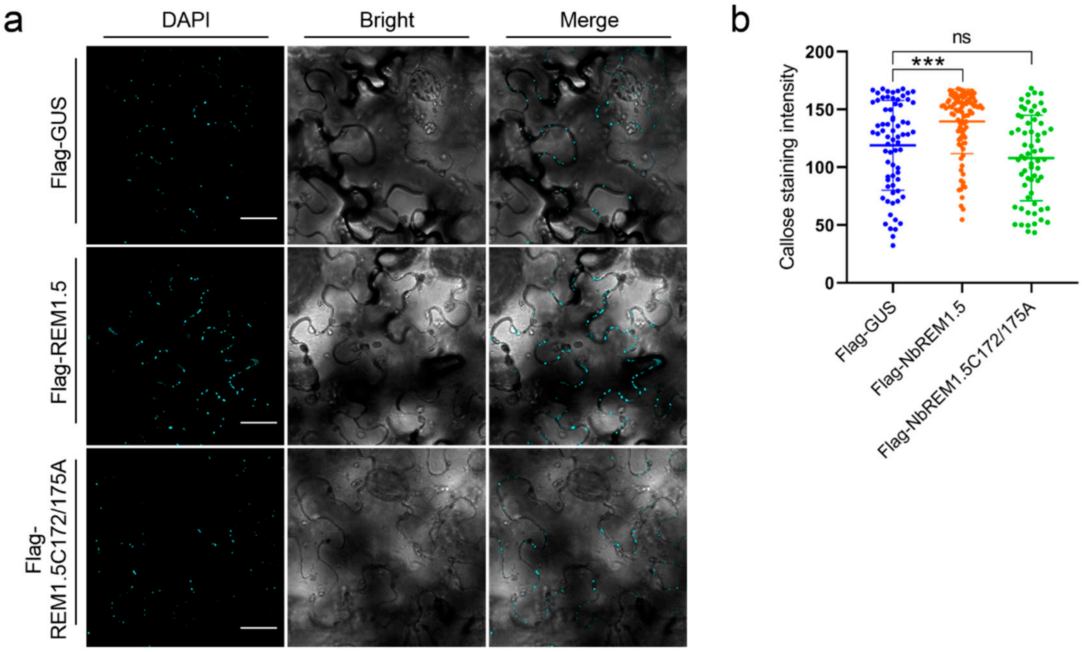

Figure S6. Callose deposition assay in *N. benthamiana* epidermal cells overexpressing Flag-GUS, Flag-NbREM1.5 or Flag-NbREM1.5-C172/175A by agroinfiltration. **(a)** Callose deposition assay by using aniline blue staining in *N. benthamiana* epidermal cell that overexpression of Flag-GUS, Flag-NbREM1.5 or Flag-NbREM1.5-C172/175A by agroinfiltration. Bar, 20μm. **(b)** Statistical analyses of callose deposition in *N. benthamiana* epidermal cell that overexpression of Flag-GUS, Flag-NbREM1.5 or Flag-NbREM1.5-C172/175A by agroinfiltration. Data are mean ± SEM (n=65). Asterisks mark significant differences according to two-tailed Student's t-test; ***$p < 0.001$; "ns", no significant difference.

Table S1 Prediction of palmitoylation sites of the nine group 1 NbREM proteins by GPS-Palm.

	Protein(ID)	Position	Peptide	Score	Cutoff
1	NbREM1.1 /Niben101Scf04804g01002.1	206	APKKLLG**C**F.	4.3	1.079
2	NbREM1.2 /Niben101Scf02910g01040.1	209	APKKLLG**C**F.	4.3	1.079
3	NbREM1.3 /Niben101Scf17127g00017.1	177	PKKPLLG**C**F.	3.533	1.072
4	NbREM1.4 /Niben101Scf15568g00037.1	172/175	APKKLLG**C**LG**C**.	4.191/1.541	1.079 /1.072
5	NbREM1.5 /Niben101Scf08587g00002.1	172/175	APKKLLG**C**LG**C**.	4.191/1.541	1.079
6	NbREM1.6 /Niben101Scf01824g00007.1	195	IPKKFLS**C**FGR	1.372	1.072
7	NbREM1.7 /Niben101Scf01655g03006.1	149	TPKKLLG**C**VG	3.719	1.079
8	NbREM1.8 /Niben101Scf00647g01005.1	195	IPKKFLS**C**FGR.	1.203	1.072
9	NbREM1.9 /Niben101Scf05118g04011.1	197	SPKKFLG**C**F	3.638	1.079

Table S2 Primers used in this study.

**qRT-PCR**	
RT-NbREM1.1/1.2-F	TTGCTGCATGGGAGAATAGTAAG
RT-NbREM1.1/1.2-R	CCTTAAGAAGATCTTCTCCACGTTTA
RT-NbREM1.3-F	ATGATGGCAGAAGAAACAGCTAAG
RT-NbREM1.3-R	ATCTCTATCAATAGATCCTCTTTTCTCAA
RT-NbREM1.4/1.5-F	GCAACTAGAAGAAAAGAAAGCAGAAT
RT-NbREM1.4/1.5-R	CAAGGCATCCAAGCAACTTCTTA
RT-NbREM1.6/1.8-F	CTCCCTCTAAACCTCCTCCTTC
RT-NbREM1.6/1.8-R	CCATCTTGTTCTTCATCTTCTCTGC
RT-NbREM1.7-F	CGCAGCTTAATAATGACAAGAAGTCTG
RT-NbREM1.7-R	TTAGGAGTTTGTCCTGTGGCGATAT
RT-NbREM1.9-F-2	CTCTCACCACCGTCGACACAC
RT-NbREM1.9-R-2	GTGTTTTCCCATCTAGCAACTGCA
RT-NbActin-F	CAATCCAGACACTGTACTTTCTCTC
RT-NbActin-R	AAGCTGCAGGTATCCATGAGACTA
**NbREMs palmitoylation, localization, and expression assays**	
GFP/FLAG-NbREM1.1-Xba1-F	ggtacccggggatcctctagaATGGCAGAAGTAGAAGCTAC
GFP/FLAG-NbREM1.1-Xba1-R	gctctgcaggtcgactctagaTCAAAAACATCCAAGGAGTTTC
GFP/FLAG--NbREM1.1-C206A-Xba1-R	gctctgcaggtcgactctagaTCAAAATGCTCCAAGGAGTTTC
GFP/FLAG-NbREM1.3-Xba1-F	ggtacccggggatcctctagaATGATGGCAGAAGAAACAGCTAAG
GFP/FLAG-NbREM1.3-Xba1-R	gctctgcaggtcgactctagaTCAAAAACATCCAAGGAGAGGTTT
GFP/FLAG--NbREM1.3-C177A-Xba1-R	gctctgcaggtcgactctagaTCAAAATGCTCCAAGGAGAG
GFP/Flag-NbREM1.5-Xba1-F	ggtacccggggatcctctagaATGGCAGAAGCAACTCCAGTATC
GFP/Flag-NbREM1.5-Xba1-R	gctctgcaggtcgactctagaTTAGCATCCAAGGCATCCAAG
GFP/Flag-NbREM1.5-C172A-Xba1-R	gctctgcaggtcgactctagaTTAGCATCCAAGTGCTCCAAG
GFP/Flag-NbREM1.5-C172/175A-Xba1-R	gctctgcaggtcgactctagaTTATGCTCCAAGTGCTCCAAGCAACTTCT
GFP/Flag-NbREM1.8-Xba1-F	ggtacccggggatcctctagaATGGGAGAAGAAGCAACTCT
GFP/Flag-NbREM1.8-Xba1-R	gctctgcaggtcgactctagaTTAGCGGCCAAAACAACTC
GFP/Flag-NbREM1.8-C195A-Xba1-R	gctctgcaggtcgactctagaTTAGCGGCCAAATGCACTC
mScarlet-Flag-Sma1-F	gggacgagctcggtacccgggATGGTTTCTAAGGGTGAAGCTGTT
mScarlet-Flag-Sma1-R	cgactctagaggatccccgggTTTTGTAAAGCTCATCCATACCTCC
GUS-Flag-Sma1-F	gggacgagctcggtacccgggATGTTACGTCCTGTAGAAACCCCA
GUS-Flag-Sma1-R	cgactctagaggatccccgggtTTGTTTGCCTCCCTGCTGC
**VIGS**	
TRV-RNA2-NbREM1.5-BamH1-F	agaaggcctccatggggatccATGGCAGAAGCAACTCCAGTATC
TRV-RNA2-NbREM1.5-BamH1-R	cgtgagctcggtaccggatccGCTTTCTTTTCTTCTAGTTGCTCCTC
